# Time dynamics of stress legacy in clonal transgenerational effects: A case study on *Trifolium repens*

**DOI:** 10.1002/ece3.8959

**Published:** 2022-05-24

**Authors:** Jiaxin Quan, Zuzana Münzbergová, Vít Latzel

**Affiliations:** ^1^ 86891 Key Laboratory of Resource Biology and Biotechnology in Western China Ministry of Education Northwest University Xi’an China; ^2^ 48311 Institute of Botany Czech Academy of Sciences Průhonice Czech Republic; ^3^ Department of Botany Faculty of Science Charles University Prague Czech Republic

**Keywords:** 5‐azacytidine, DNA methylation, epigenetic memory, stress legacy persistence

## Abstract

Stress can be remembered by plants in a form of stress legacy that can alter future phenotypes of previously stressed plants and even phenotypes of their offspring. DNA methylation belongs among the mechanisms mediating the stress legacy. It is however not known for how long the stress legacy is carried by plants. If the legacy is long‐lasting, it can become maladaptive in situations when parental–offspring environment do not match. We investigated for how long after the last exposure of a parental plant to drought can the phenotype of its clonal offspring be altered. We grew parental plants of three genotypes of *Trifolium repens* for five months either in control conditions or in control conditions that were interrupted with intense drought periods applied for two months in four different time slots. We also treated half of the parental plants with a demethylating agent (5‐azacytidine, 5‐azaC) to test for the potential role of DNA methylation in the stress memory. Then, we transplanted parental cuttings (ramets) individually to control environment and allowed them to produce offspring ramets for two months. The drought stress experienced by parents affected phenotypes of offspring ramets. The stress legacy resulted in enhanced number of offspring ramets originating from plants that experienced drought stress even 56 days before their transplantation to the control environment. 5‐azaC altered transgenerational effects on offspring ramets. We confirmed that drought stress can trigger transgenerational effects in *T*. *repens* that is very likely mediated by DNA methylation. Most importantly, the stress legacy in parental plants persisted for at least 8 weeks suggesting that the stress legacy can persist in a clonal plant *Trifolium repens* for relatively long period. We suggest that the stress legacy should be considered in future ecological studies on clonal plants.

## Graphical abstract

Environmental stress can be remembered by plants in a form of “stress legacy.” We investigated for how long can a parental plant “remember” that it experienced a stress and pass the memory to its clonal offspring.

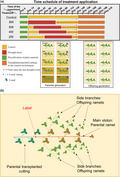

## 1 INTRODUCTION

An increasing body of studies demonstrate that plants’ exposure to different kinds of stresses in the past can affect their responses to the same and/or different stresses in the future and eventually prepare them to respond rapidly and/or adaptively to forthcoming stressful events (Bruce et al., 2007; Ding et al., 2013; Iwasaki & Paszkowski, 2014; Li et al., 2014, 2019; Ramírez et al., 2015). Such a phenomenon is commonly called “stress legacy”, “stress memory,” or “priming.” In some cases, the stress experience can be passed to further generation(s) and affect thus offspring growth and response to the stress despite no direct exposure to the stress (Cullins, 1973; Molinier et al., 2006; Monneveux et al., 2013; Shock et al., 1998; Trewavas, 2014). Such transgenerational effects can allow for rapid adaptation to environmental condition if offspring environment resembles parental conditions (Boyko & Kovalchuk, 2011; Crisp et al., 2016; González et al., 2017; Latzel et al., 2014; Latzel & Klimešová, 2010; Mirouze & Paszkowski, 2011; Puy et al., 2020).

An intriguing question is for how long is the stress legacy affecting the phenotypes of offspring? If the stress legacy has physiological and/or phenotypic consequences on the offspring and is maintained over long period by the parental plant, it could easily become maladaptive in situations when stress events are rare or even absent. On the other hand, if the stress legacy is kept only for a very short time it can have limited if any transgenerational effects and thus potentially no role in transgenerational adaptation. In other words, in order for memory to be advantageous to plants, plants must balance between creating and keeping memory and being able to reset the memory (Crisp et al., 2016). Information on the experienced stress can be stored in the form of epigenetic variation (Bruce et al., 2007; McIntyre & Strauss, 2014; Pascual et al., 2014; Richards et al., 2017). It has been shown that environmentally induced epigenetic variation can be transmitted to offspring generations (e.g., González et al., 2018; Verhoeven & van Gurp, 2012; Verhoeven et al., 2010) and can be gradually lost after several sexual or asexual generations in the absence of the triggering environmental stress (Jiang et al., 2014; Shi et al., 2019). However, the knowledge of temporal dynamics of the stress legacy on offspring phenotype remains limited.

The dynamic of environmental stress can be operating at time scales ranging from several days to few weeks. For example, in the central European context, a relatively wet spring is often followed by several weeks of drought in summer ending with a wet period in the autumn (www.chmi.cz). A new clonal offspring will thus likely experience conditions different from those of the parental plant. Nonetheless, we still do not know whether such environmental dynamics is accounted for in the stress legacy dynamics in clonal plants adapted to such environments.

Drought is one of the main threats affecting plant growth, as water deficit affects plants at all levels from molecular, cellular, organ to the whole body (Avramova, 2015; Li et al., 2014; Li & Liu, 2016; Tombesi et al., 2018). Studies have shown that plants that experienced repeated cycles of drought stress exhibited both transcriptional and physiological responses during a subsequent drought stress that were absent in plants without previous drought experience (Ding et al., 2012, 2014; Virlouvet et al., 2018). It has been also shown that the memory of drought can be passed to (a)sexual offspring in *Oryza sativa*, *Trifolium repens*, *Arabidopsis thaliana*, and *Zea mays* (Ding et al., 2012, 2014; González et al., 2016; Li et al., 2019; Virlouvet et al., 2018) and can even be adaptive, that is, offspring of stressed parents overcome the stress better, that is, has higher overall fitness, than a naïve offspring (González et al., 2017). Clonal plants usually prefer wet habitats (Klimeš et al., 1997; van Groenendael et al., 1996) making them particularly vulnerable to drought events that should increase in their frequency and severity in the near future (Dai, 2012; Sherwood & Fu, 2014).

Clonal plants may have greater ability to pass epigenetic information to asexual offspring than non‐clonal plants to sexual offspring because of the lack of meiosis during clonal reproduction (Douhovnikoff & Dodd, 2015; González et al., 2016; Latzel & Klimešová, 2010; Latzel & Münzbergová, 2018; Münzbergová et al., 2019; Paszkowski & Grossniklaus, 2011; Verhoeven & Preite, 2014). This makes clonal plants an ideal system for studying various ecological and evolutionary aspects of transgenerational stress memory in plants. Our previous studies on a clonal herb *Trifolium repens* have shown that it can develop genotype‐specific drought stress legacy that is partly enabled by epigenetic mechanism, in this case by DNA methylation (González et al., 2016, 2018). We have also shown that the stress legacy can be adaptive, that is, offspring ramets of parents that experienced drought responded to the drought better and produced more biomass, than naïve offspring (González et al., 2017). The legacy is translated into altered growth of offspring ramets in comparison to plants without the legacy (González et al., 2016, 2017).

Here, we built on our previous studies on *T*. *repens* and tested for how long from the last exposure of a parental plant to the drought can phenotype of its clonal offspring be affected and whether the offspring phenotype alteration is co‐facilitated by DNA methylation. We tested the following hypotheses: (1) Drought stress is altering the growth (for example, reduced biomass or number of branches) of parental ramets. (2) This alteration triggers drought‐stress legacy that affects phenotype of offspring ramets but is time‐limited and is lost after a certain period since the last drought event. (3) The drought stress legacy is facilitated by DNA methylation. Testing these hypotheses should enable us to put the phenomenon of transgenerational effects into a time frame context, which may improve our understanding of ecological and evolutionary consequences of transgenerational effects in clonal plants.

## 2 MATERIALS AND METHODS

### 2.1 Plant material

We used *Trifolium repens* (Fabaceae) as the model in our study. It is a rapidly growing polycarpic perennial herb widely distributed in a variety of grasslands and pastures differing in soil type, nutrient level, and soil humidity (Burdon, 1983).

**FIGURE 1:**
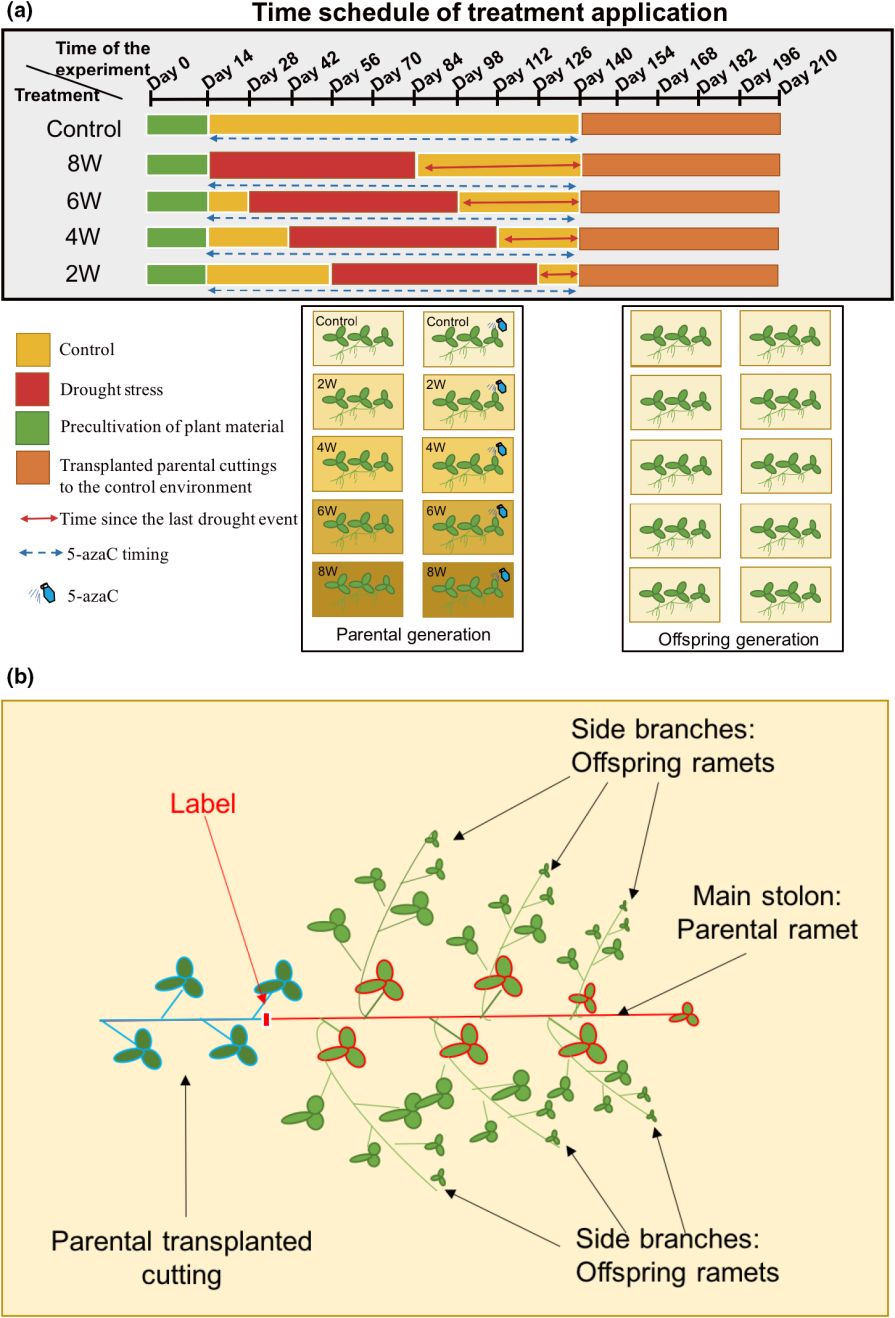
(a) Time schedule of the experiment. (b) Idealized scheme of *Trifolium repens* plant developed after transplantation of parental cutting to a control environment. Label: marked position of apical end of transplanted parental ramet. This enabled determination of parental ramet that developed prior transplantation to the control environment

In most studies, each phytomer of *T*. *repens* that consists of a node, internode, leaf, axillary bud, and two nodal root initials is considered as a ramet (Gómez et al., 2007; Hay et al., 2001). However, similarly to our previous studies on the species (González et al., 2016, 2017, 2018), we decided to apply a more conservative approach and consider offspring ramets only the side branches produced by the elongating main stolon, that is, parental ramet. The monopodial growth style of *T*. *repens* means that every stolon elongates along its main axis by producing new phytomers within which resource and information flow is not restricted. On the other hand, the side branches that are produced by axillary buds of the main stolon are more independent from the main stolon because their connection to the main stolon is limited and not permanent, which results in more limited resources and information exchange among the main stolon and side branches. In other words, the growth of side branches is more independent of the physiological state of the main stolon. Such a conservative approach provides us confidence that we can consider potential observed environmental effects to be truly transgenerational and ecologically relevant. See also Figure 1 for a description of parental and offspring clonal generations considered in our study.

We collected three cuttings taken from at least 50 m distance from a mesophilous meadow of the park at the Institute of Botany, Průhonice, Czech Republic, to ensure that the three cuttings were of different genotypes but had similar growing conditions as well as growing history. We vegetatively propagated them for four months in the experimental garden prior the main experiment.

### 2.2 Study design

We conducted the experiment in a greenhouse at the Institute of Botany, Průhonice, Czech Republic, with controlled temperature and light regime from October 7, 2019 to May 4, 2020 (210 days in total). The greenhouse had controlled temperature (23/18°C day/night) and light regime (12‐/12‐h light/night cycle). The experiment was divided into two parts. The first consisted of stress legacy induction in the parental generation, the second was designed to test for how long the parental plant carries legacy of the drought stress that affects clonal offspring generations.

#### 2.2.1 First phase–drought stress application

We created 120 standardized unbranched cuttings (parental ramets) from the pre‐cultivated plant material (three genotypes, 40 cuttings per genotype) of *T*. *repens*. Each cutting consisted of three nodes with apical end and was planted individually into a tray 30 × 40 × 8 cm filled with standardized soil (Trávníkový substrát, AGRO CS a.s., Rikov, Czech Republic, mixture of sand, compost, and peat, 75% mass water holding capacity). After transplantation of parental ramets, we kept all plants in control conditions (regular watering) for two weeks to allow recovery and successful rooting. Afterward, we randomly assigned plants to five treatment combinations: control (n=8 per genotype), plants were watered regularly to keep the soil constantly moist during the whole cultivation period and 4 drought‐stress treatments. The plants were grown for 5 months in selected conditions. Plants assigned to drought stress treatment experienced control conditions interrupted with drought periods (watered only when leaves were wilting) that lasted for 10 weeks but in different time slots (2 weeks difference among the slots, see Figure 1). In the first group (*n* = 8 per genotype), the drought treatment ended 8 weeks before establishment of the Offspring generation part (further referred to as 8W group, see also Figure 1). In the second group (*n* = 8 per genotype), drought ended 6 weeks before establishment of the Offspring generation part (further referred to as 6W group). In the third group (*n* = 8 per genotype), drought ended 4 weeks before establishment of the Offspring generation part (further referred to as 4W group). Finally, in the fourth group (*n* = 8 per genotype), drought ended 2 weeks before establishment of the Offspring generation part (further referred to as 2W group). The drought stress was implemented by watering a plant with 200 ml of water only when the plant showed significant drought stress response, that is, most leaves wilting. The water volume was determined by a pilot study to sufficiently moistened the soil and ensured that the next drought pulse occurs within 4–7 days. During the 10‐week drought period, plants were watered approximately 10 times. The control plants received 8 × more water than the drought‐stressed plants during the drought period (watered 2 × more often with 4 × more water volume at each watering occasion). The same level of watering as in controls was maintained in the drought‐stressed plants outside the drought period. The first phase was terminated 140th day of the experiment.

#### 2.2.2 5‐azacytidine application

To test for the role of DNA methylation in the stress memory induced by drought, we applied 5‐azacytidine demethylating agent on half of the parental plants, the remaining plants were sprayed with the same volume of pure water. 5‐azacytidine (further referred to as 5‐azaC) reduces the global cytosine methylation level of treated plants, and it has been successfully applied to demonstrate the role of plant epigenetic memory in plant adaptation to stress (e.g., Boyko et al., 2010; González et al., 2016). 5‐azaC can be toxic to plants and thus some growth responses of plants can be consequences of the toxicity rather than the alteration of DNA methylation. The unwanted side effects of 5‐azaC are, however, related almost exclusively to situations when plants are germinated in 5‐azaC solution (Puy et al., 2018). Foliar applications of 5‐azaC bypasses most of the negative effects on plant growth but keeps its demethylating efficiency at comparable levels to germination plants in 5‐azaC solution (Puy et al., 2018). We subjected half of the parental plants to 5‐azaC treatment (4 plants per genotype and treatment) to alter their epigenetic memory. We regularly sprayed plants with 100 μmol solution of 5‐azaC (Sigma‐Aldrich, Praha, Czech Republic) every fourth day, which resulted in 32 spraying events. The first application was on October 21, 2019, that is, 14 days after setting the experiment (the day of start of the first drought treatment), and with the last application at the time of the termination of the last drought treatment (February 10, 2020, 126th day of the experiment). We sprayed the plants in early morning to ensure that plants had open stomata and the solution of 5‐azaC could therefore be easily absorbed by the leaves. We did not measure the level of demethylation achieved by the 5‐azaC treatment in this study. However, in our previous study on the same species, by spraying plants eleven times with 50 μmol solution of 5‐azaC (i.e., half concertation and a third of spraying events than used in this study) resulted in overall reduction in methylation by 4.48% (González et al., 2016). Therefore, we are confident that the application of 5‐azaC was effective in this study and resulted in reduction of overall DNA methylation level of treated plants. However, we cannot exclude the scenario that plants experiencing drought can react to the 5‐azaC differently than plants experiencing control conditions.

#### 2.2.3 Second phase—testing of stress legacy dynamics

On day 140 of the experiment, we created a single standardized parental cutting consisting of four nodes and apical end from each individual (40 cuttings per genotype, 120 cuttings in total) and transplanted them individually to similar trays filled with the same substrate as in the first phase. The remaining above ground biomass of parental plants (further referred to as “parental biomass”) was harvested, dried at 80°C for 48 h, and weighed. By creating a cutting, we ensured that the newly growing clone had no connection to the original parental plant from the first phase. Thus, the new emerging clone could not receive any signals from the parental plant that experienced the drought and all phenotypic differences potentially detected on the newly emerging clone can be ascribed to stress legacy mechanisms carried by the transplanted cutting.

We cultivated the transplanted plants in a greenhouse under control condition for 10 weeks (from Day 140 to Day 210 of the experiment). We labeled the apical end of each transplanted cutting to be able to identify the end of parental (transplanted) ramet that had developed before transplantation and the new parts that have developed after transplantation (see Figure 1b). During the 10 weeks’ period, we recorded the length of the main stolon (parental ramet), number of nodes of the parental ramet, and number of side branches, that is, offspring ramets every week (10 times in total). At the end of the experiment (ten weeks after establishment of the Offspring generation), we record the number of side branches (i.e., offspring ramets) produced by the elongating transplanted parental ramet. All clones consisted of interconnected ramets at the end of the study. We harvested above‐ground biomass separated in parental ramet (main stolon was divided into parts developed before and after transplantation) and offspring ramets (side branches) that had developed after transplantation, dried them at 80°C for 48 h and weighed. The mean offspring biomass was calculated as total offspring biomass divided by the number of side branches.

In a subset of randomly chosen plants, we also checked the Rhizobia colonization of roots. We did not find any established relationship in the 10 plants, which confirmed our previous experience with the species that the Rhizobia colonization is rare under our growing conditions.

### 2.3 Statistical analyses

We tested the effect of genotype (genotype A, genotype B, genotype C), time since the last drought (2W, 4W, 6W, 8W where W means week, and Control), 5‐azaC application (control, 5‐azaC) and their interactions on parental biomass of the first phase, mean offspring biomass developed in the second phase and final number of branches. Using generalized linear models with Poisson distribution for number of branches and Gaussian distribution for the other two variables using glm function in R3.5.1 (R Core Team, 2018). For the variables measured in the second phase, we also included initial size of the cutting and its interaction with other variables into the models. Because our models were very complex, we use AIC to select the optimal model (only main effects or also double and triple interactions and inclusion of initial size or not, Table S1). The effect of each predictor in the optimal model was assessed after accounting for all the other predictors in the model, within a given level of complexity. So, the model first included only all main effects, and we tested the effect of each main effect after accounting for all the other main effects. Then the second‐order interactions have been tested after accounting for all the main effects and all the other second‐order interactions etc. We used duncan.test function in the agricolae package in R (de Mendiburu et al., 2020) to perform the post hoc tests in case of significant effects. To meet the assumptions of homoscedasticity and normality, the biomass data were log transformed prior to analyses.

To explore growth dynamics of the plants, we used mixed‐effect models with genotypes, time since last stress, 5‐azacytidine application (control, 5‐azaC), time of measurement (10 measurements), and initial size and all their interactions as predictors and individual code as a random factor. The dependent variables were main stolon length, offspring branch number (following Poisson distribution) and node number (following Poisson distribution) in the second phase. As above, we used AIC to select the optimal level of complexity of the model and use the same approach to perform the tests. As the results of the tests included time were largely similar to result of the above overall test, the tests with time is only shown in the Tables S3–S5 and Figures [Link], [Link], [Link], [Link], [Link], [Link]. The mixed models were performed using lme4 package (Bates et al., 2015) in R3.5.1.

## 3 RESULTS

### 3.1 Parental plants of the first phase

Parental biomass differed among the genotypes (mean ± SE, genotype A: 24.50 g ± 3.87; genotype B: 17.87 g ± 2.83; genotype C: 24.21 g ± 3.83) and was affected by the time period since the last drought (Table 1). Control plants were the biggest, whereas the plants that received drought treatment were on average half the size of control plants. Parental plants with the last drought treatment 8 weeks before transplantation were the biggest and parental plants that received last drought 2 weeks before transplantation were the smallest among the plants that received the drought stress (Figure S1).

**TABLE 1:** Effects of genotype, time since last drought (2W, 4W, 6W, 8W, and Control), and 5‐azaC treatment (control *versus* 5‐azaC) on parental biomass and mean offspring biomass developed in the second phase and final side branch number of *Trifolium repens* based on the optimal model selected based on AIC (Table S1)

	*df*	Parental biomass^a^			Mean offspring biomass^a^			Side branch no.^b^		
		*F*	*p*	*R* ^2^ Value	*F*	*p*	*R* ^2^ Value	Dev.	Pr(Chi)	*R* ^2^ Value
Genotype	2	**29.08**	**<.001**	.074	0.09	.917	.001	**10.96**	.**004**	.050
Time since last drought (T)	4	**153.45**	**<.001**	.781	1.27	.289	.031	**32.58**	**<.001**	.149
5‐azaC	1	2.29	.133	.003	**23.12**	**<.001**	.139	**14.32**	**<.001**	.065
Genotype × T	8	–	–	–	1.73	.102	.084	–	–	
Genotype × 5‐azaC	2	–	–	–	**3.37**	.**039**	.041	–	–	
T × 5‐azaC	4	–	–	–	*2.43*	.*053*	.059	–	–	
Genotype × T × 5‐azaC	8	–	–	–	**2.14**	.**040**	.103	–	–	

Note

Values for *p* < .05 are in bold. Marginally significant (*p* < .1) in italics. – indicates the factor not included in the best model.

*R* ^2^ values are expressed based on Sum Sq/deviance of the model for models with Gaussian/Poisson distribution.

^a^ Log transformed following Gaussian distribution.

^b^ Follows Poisson distribution.

### 3.2 Offspring plants of the second phase

**FIGURE 2:**
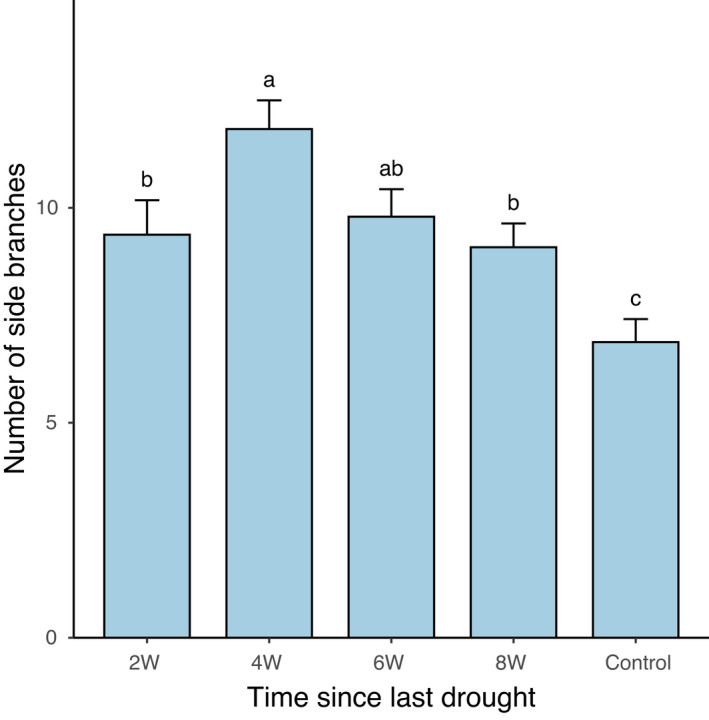
Effect of time since the last drought event (2W, 4W, 6W, 8W *versus* Control) experienced by parental ramets on the production of side branches (clonal offspring) of *Trifolium repens*. Means and SE are shown. Columns sharing the same letter are not significantly different from each other at *p* < .05

The number of side branches (offspring ramets) significantly differed among genotypes (Table 1). The number of side branches was also significantly affected by the time period since the last drought (Table 1). Plants of parents that experienced drought before transplantation produced more branches than control plants irrespective of the drought timing (Figure 2).

### 3.3 The effect of 5‐azacytidine on Offspring generation

**FIGURE 3:**
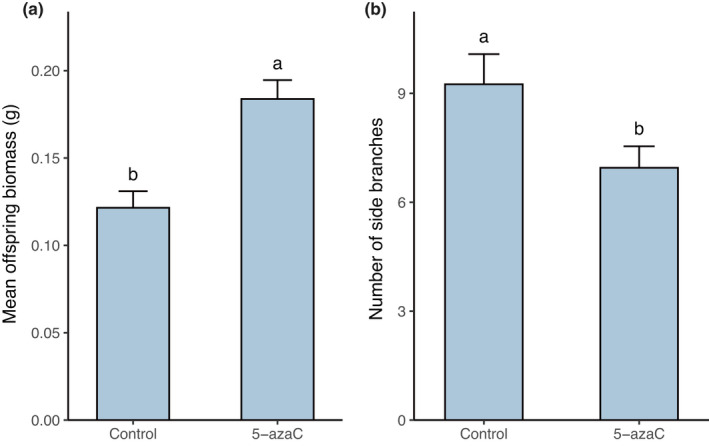
Effect of 5‐azaC on the mean offspring biomass (a) and number of side branches (b) (offspring) of *Trifolium repens*. Means and SE are shown. Columns sharing the same letter are not significantly different from each other at *p* < .05

**FIGURE 4:**
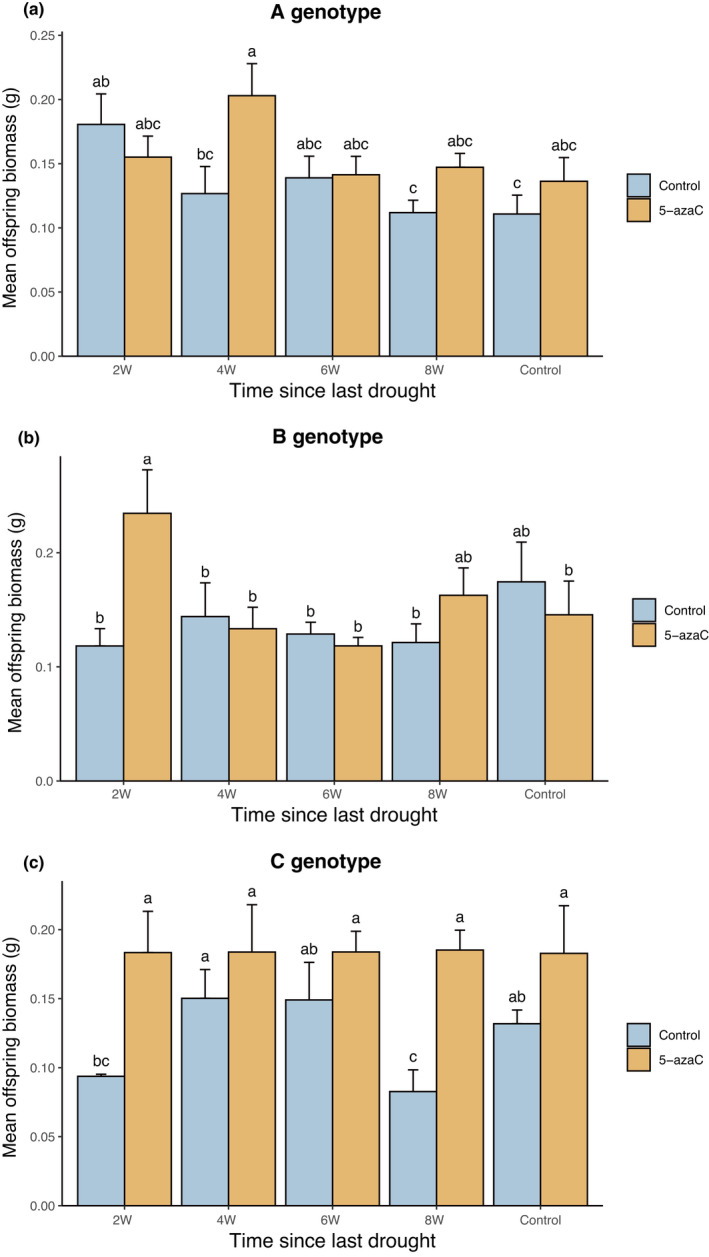
Interactive effect of time since the last drought event (2W, 4W, 6W, 8W *versus* Control) and 5‐azaC on mean offspring biomass of 3 genotypes (A genotype, B genotype, and C genotype) of *Trifolium repens*. Means and SE are shown. Columns sharing the same letter are not significantly different from each other at *p* < .05

Application of 5‐azaC on parental plants in the first phase of the study consequently increased the mean offspring biomass and reduced number of side branches in transplanted plants of the second phase (Table 1, Figure 3a,b), but did not have a main effect on the other measured variables in transplanted plants. The interaction between 5‐azaC and time since the last drought was marginally significantly altered mean offspring biomass. The mean offspring biomass of parents that experienced the last drought event 2 and 8 weeks before transplantation significantly increased compared to offspring of control parents (Figure S2). The effect of 5‐azaC was strongly genotype dependent (Table 1). In genotype A, the mean offspring biomass of parents that experienced the last drought event 4 weeks before transplantation significantly increased after 5‐azaC application. In genotype B, significant effect of application of 5‐azaC on parental plants was detected in plants that experienced last drought event two weeks before transplantation. In genotype C, plants which experienced the last drought event 2 and 8 weeks before transplantation were significantly bigger after 5‐azaC application when compared to offspring of control parents (Table 1, Figure 4).

## 4 DISCUSSION

Our study investigated whether drought stress in the parental generation triggers transgenerational effects in a clonal plant *Trifolium repens*, and if so, for how long from the last drought event the stress legacy in parental plant persists and affects the phenotype of its clonal offspring. We hypothesized that the phenotypic consequences of transgenerational effects should be gradually erased with the increasing time since the last drought event. This prediction assumes that the long‐term phenotypic consequences of transgenerational effects should not be beneficial in situation when the drought stress is infrequent, time‐limited, or even absent for a long period (Jiang et al., 2014; Lukic et al., 2020; Shi et al., 2019).

Results of our study are not in agreement with our predictions. We found that drought stress was detectable on the number of created offspring ramets even 8 weeks after the last drought experienced by parents in all genotypes. Our results thus suggest that the legacy of drought stress in a parental plant can last for at least 8 weeks (we did not test longer period because drought events simulated in our study cannot be expected to last more than few weeks in the Central European context) and trigger transgenerational effects that are affecting offspring phenotypes of *T*. *repens*. This contradicts our prediction that the role of transgenerational effects should be gradually erased with the increasing time since the last drought event because they could easily become maladaptive in situations when stress events are rare or even absent. On the other hand, the long‐lasting transgenerational effects due to the drought resulted in increased number of offspring ramets produced by parental ramets that experienced drought. This suggests that the negative effect of the drought on parental biomass can be to some degree compensated in the offspring generation. In other words, the stress legacy can provide plants with other advantage than only better coping with future stress. Hence, even the long‐lasting stress legacy may not be maladaptive as long as it provides offspring with other benefits. These findings are to some degree in line with our previous study where we showed that particular intensity of drought stress in parental generation can increase offspring growth and biomass, whereas different levels of drought result in reduced biomass of offspring ramets (González et al., 2016).

Some studies showed that the environmentally induced epigenetic variation can be heritable among several (a)sexual generations in the absence of the triggering stress (Verhoeven et al., 2010; Xu et al., 2016). Shi et al. (2019) found that the environmentally induced epigenetic variation is progressively degrading over 10 clonal generations (10 offspring ramets created from the establishment of the study) when cultivating *Alternanthera philoxeroides* in a common environment. These studies however were focused only on molecular mechanisms and did not test the phenotypic consequences of environmentally induced epigenetic variation in plants. Despite that, they provided important evidence that the environmentally induced epigenetic change can be heritable in certain cases (and species) and is carried by several (a)sexual generations. In our study, we tested the role of DNA methylation on transgenerational effects indirectly via alteration of DNA methylation of half of the plants with 5‐azacytidine (5‐azaC). Our results outlined that DNA methylation was likely involved in the observed transgenerational effects as the effect of parental drought on mean offspring biomass was changed in plants treated with 5‐azaC in comparison to plants of the same stress history but not treated with 5‐azaC. Interestingly, 5‐azaC did not alter growth of control plants (see Figure 4 and Figure S2), which supports our conclusion that the application of 5‐azaC interacted with epigenetic memory on the drought stress.

The genotype specificity of the role of 5‐azaC on transgenerational effects observed in mean offspring biomass (Figure 4) is in line with other studies demonstrating that epigenetic variation can be highly genotype dependent (Becker et al., 2011; Bossdorf et al., 2008; Li et al., 2012; Richards, 2006). Alternatively, potential structural and/or morphological differences among genotypes could lead to different levels of absorption of the 5‐azaC and thus in different efficiency of demethylation of DNA. It should be also noted that the stress legacy can also be ascribed to mechanisms other than epigenetics, such as hormonal signaling or other metabolites involved in stress signaling (Hilker & Schmülling, 2019) that could be present in transplanted parental ramets.

In our study, we simulated an environment that was repeatedly desiccated during summer season, that is, periods with sufficient water supply were interrupted by periods of water shortage. This particular setting triggered stress legacy that lasted at least for 8 weeks in the three genotypes of *T*. *repens*. Of course, it is intuitive that other scenarios with different timing and/or severity of a stress could trigger different legacy effects that can have even contrasting phenotypic consequences on the offspring generation. For instance, in our previous research on the same species, we observed that the stress legacy is established only if the drought lasts for a certain period. We found that the drought stress can trigger transgenerational effects if it lasted for 10 weeks but not for 4 months (González et al., 2016). This phenomenon needs to be investigated in more detail to get better idea about the role of environmental stress, its intensity and duration on induction and temporal dynamics of transgenerational effects in plants.

Previous studies investigated the role of duration or intensity of environmental stress on induction of transgenerational effects (e.g., Boyko et al., 2010; González et al., 2016; Racette et al., 2019; Rahavi & Kovalchuk, 2013a, 2013b; Verhoeven & van Gurp, 2012) but did not consider the temporal dynamics of the stress legacy in plants. A study by González et al. (2017) showed that drought in parental generation can trigger adaptive transgenerational effects in *T*. *repens*, that is, offspring performed better in drought if their parents also experienced drought in comparison to offspring of naïve parents. However, the adaptive transgenerational effects were demonstrated on offspring of parents that experienced drought period very recently before transplantation to new environment, which may ecologically be a rather rare scenario. It is possible that documented patterns of transgenerational effects can be only snap shots in time, which can result in overestimation or underestimation of ecological and evolutionary aspects of transgenerational effects in plants.

## 5 CONCLUSION

Based on our results of the current as well as previous studies (i.e., González et al., 2016, 2017), we argue that the next step in upcoming research should be involvement of the temporal dynamics of the stress legacy from the perspective of stress duration and the time when the stress occurred in studies on clonal transgenerational plasticity. This can help us not only better understand ecological and evolutionary aspects of the transgenerational effects in clonal plants but could also improve our predictions of plant responses to future climatic conditions. More detailed insights into molecular (epigenetic) and biochemical mechanisms involved in the stress legacy would also considerably improve our understanding of the stress legacy mechanisms in clonal plants. Although we focused on clonal generations, similar aspects of temporal dynamics of stress legacy can be expected for sexually derived individuals.

## AUTHOR CONTRIBUTIONS

**Jiaxin Quan:** Conceptualization (equal); Data curation (equal); Methodology (equal); Writing – original draft (equal); Writing – review & editing (equal). **Zuzana Münzbergová:** Data curation (equal); Methodology (supporting); Supervision (equal); Writing – original draft (supporting); Writing – review & editing (supporting). **Vít Latzel:** Data curation (equal); Funding acquisition (equal); Methodology (equal); Resources (equal); Supervision (lead); Writing – original draft (equal); Writing – review & editing (lead).

## CONFLICT OF INTEREST

The authors declare no conflicts of interest.

## Supporting information

Fig S1

Fig S2

Fig S3

Fig S4

Fig S5

Fig S6

Fig S7

Fig S8

Table S1–S5

## Data Availability

Data available at Dryad https://doi.org/10.5061/dryad.s4mw6m95f.
