# Supplementary figures and images for: Time dynamics of stress legacy in clonal transgenerational effects: A case study on Trifolium repens
Source: Ecol Evol. 2022 May 24;12(5):e8959. doi: 10.1002/ece3.8959 (PMC9130644; doi:10.1002/ece3.8959)

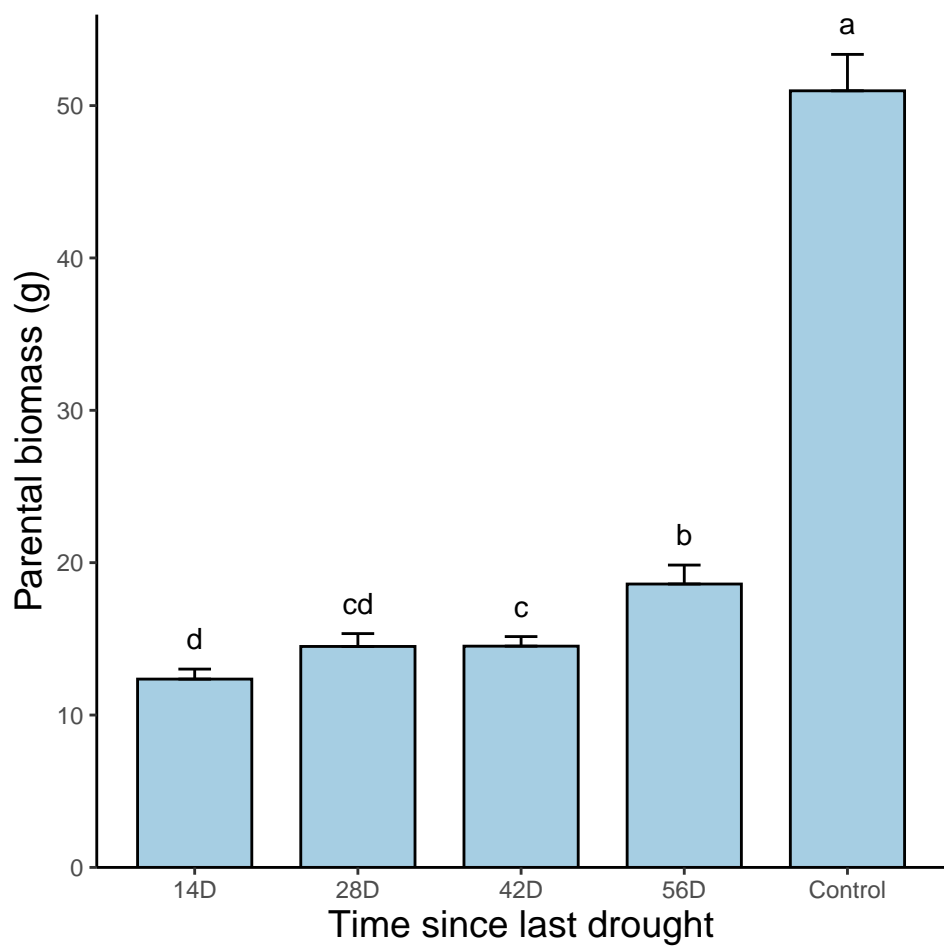

Supplement: Supplementary file 1 — Fig S1 [file ECE3-12-e8959-s004.pdf]

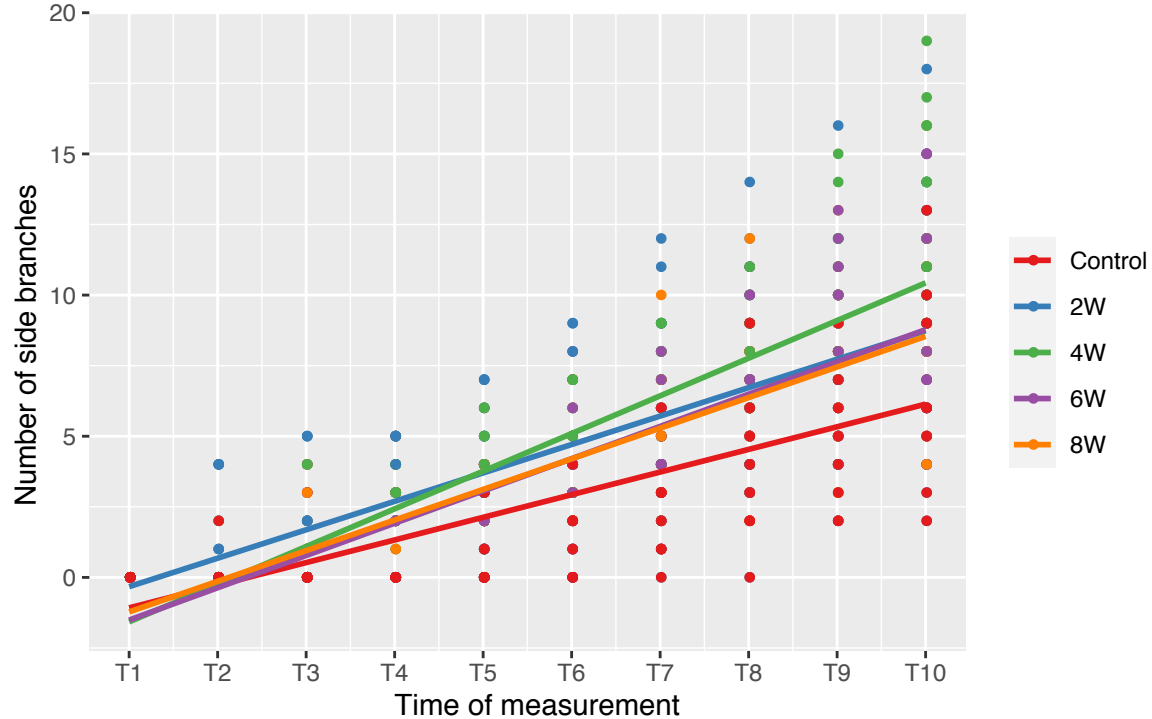

Supplement: Supplementary file 3 — Fig S3 [file ECE3-12-e8959-s005.pdf]

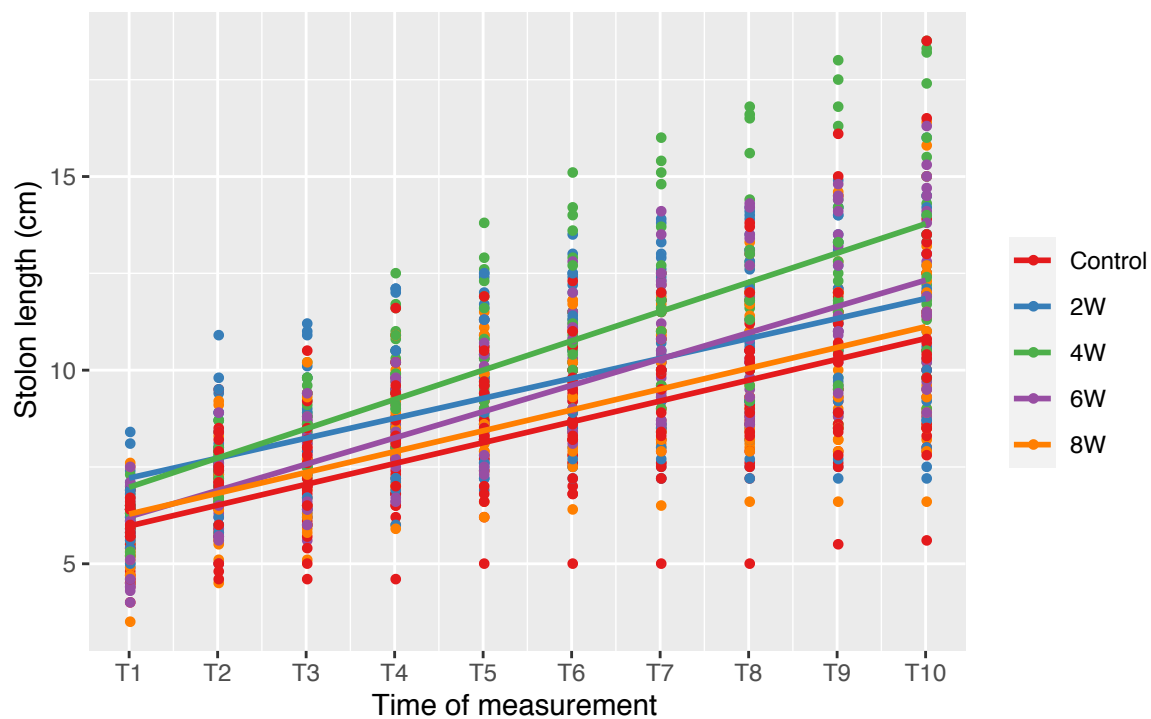

Supplement: Supplementary file 4 — Fig S4 [file ECE3-12-e8959-s002.pdf]

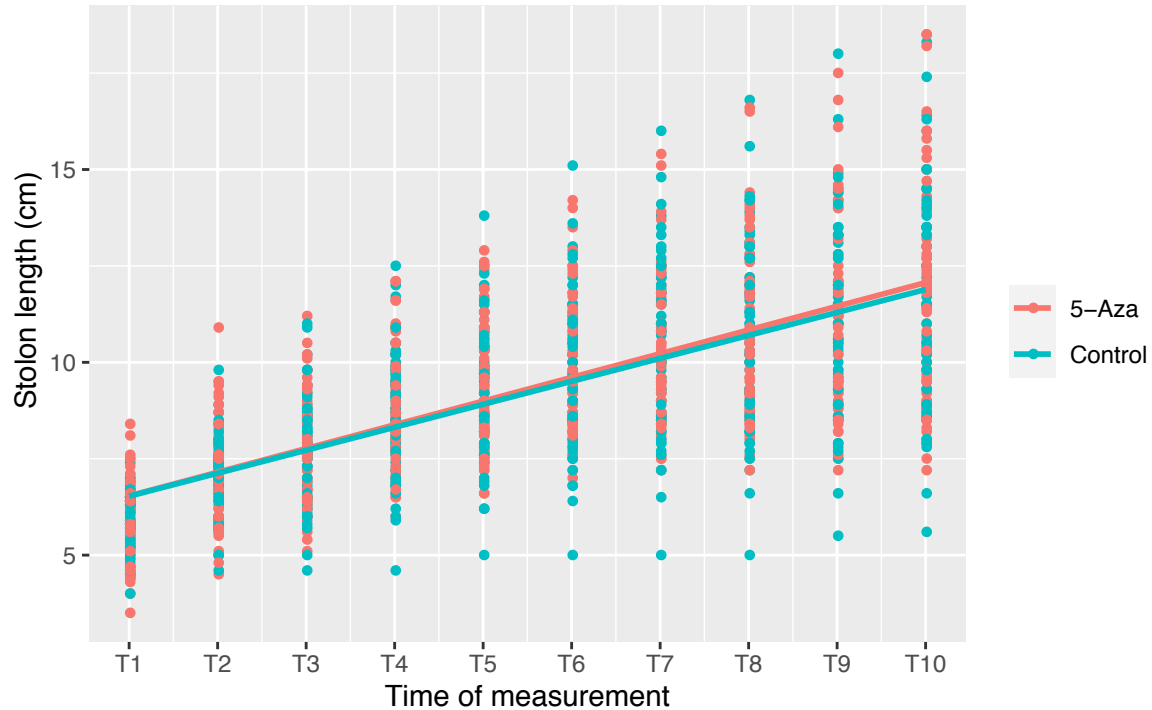

Supplement: Supplementary file 5 — Fig S5 [file ECE3-12-e8959-s009.pdf]

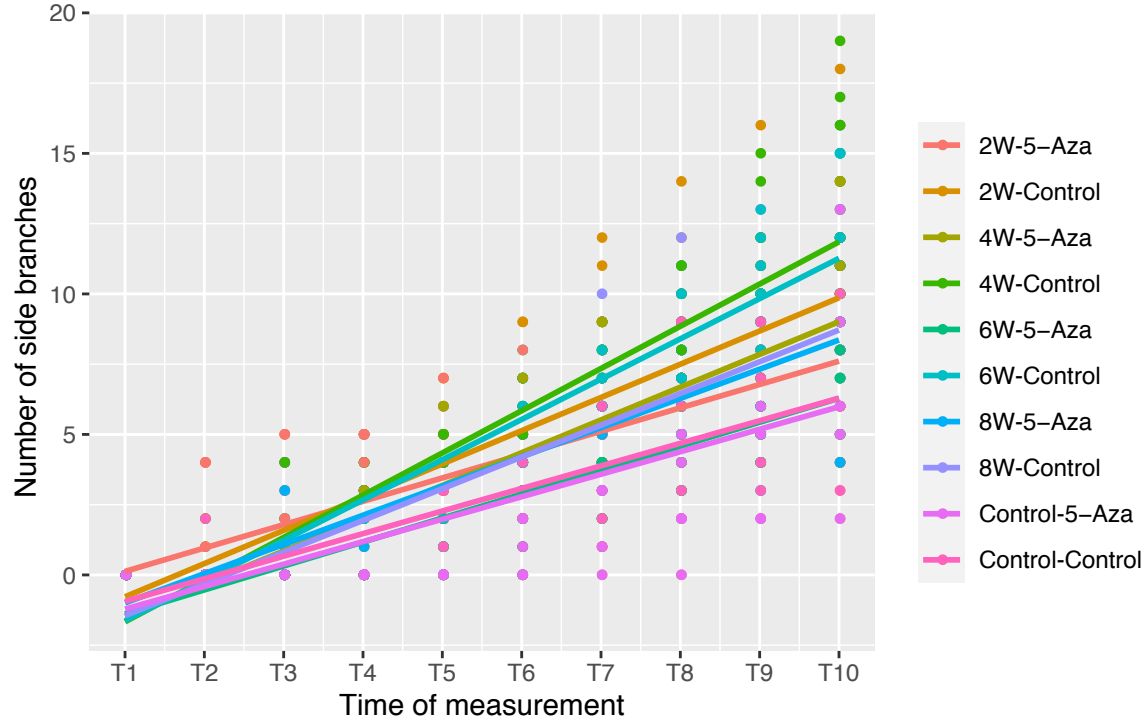

Supplement: Supplementary file 6 — Fig S6 [file ECE3-12-e8959-s007.pdf]

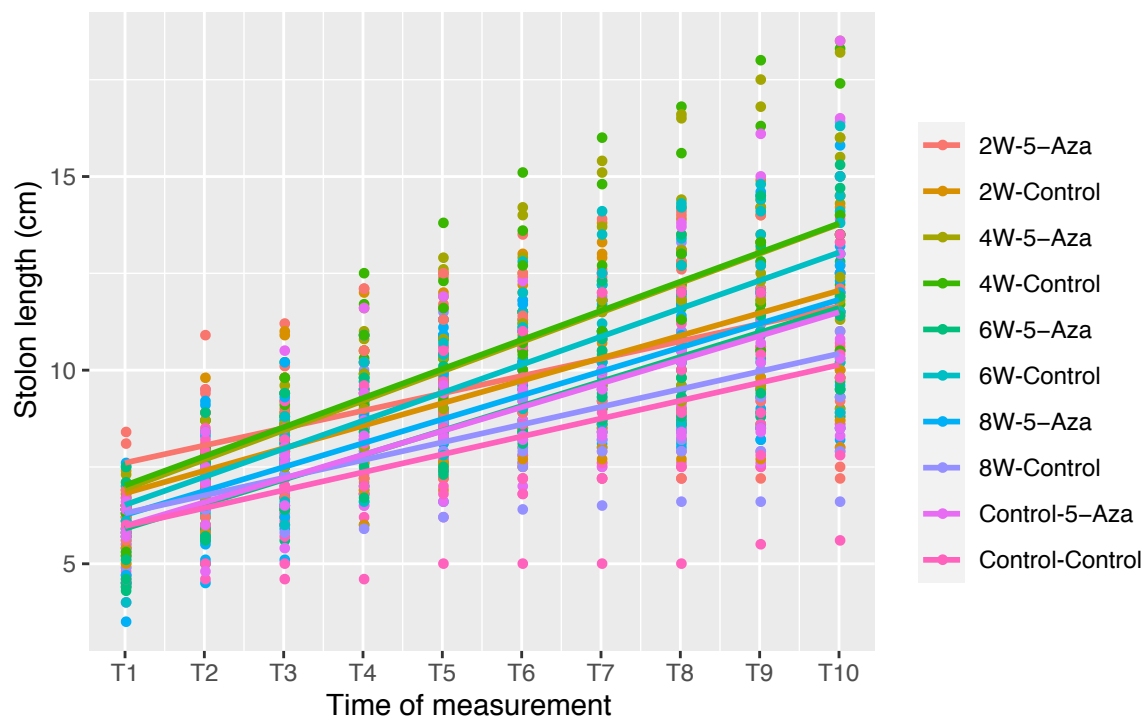

Supplement: Supplementary file 7 — Fig S7 [file ECE3-12-e8959-s008.pdf]

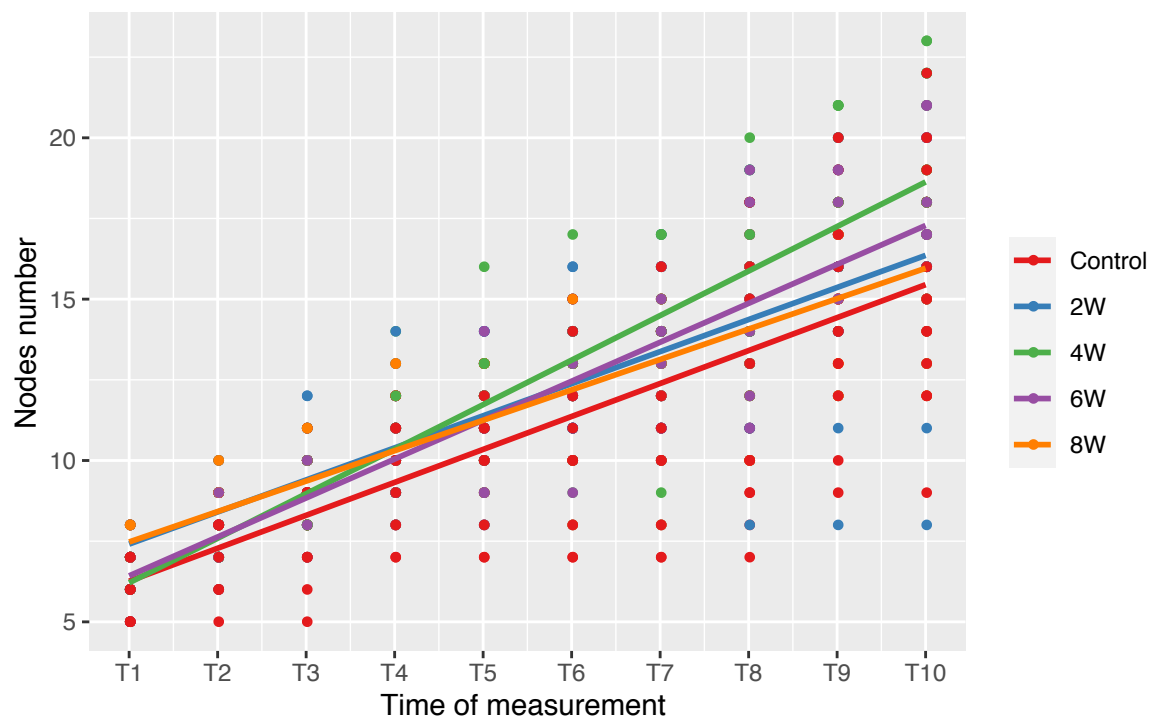

Supplement: Supplementary file 8 — Fig S8 [file ECE3-12-e8959-s001.pdf]
