# Supplementary material for: Time dynamics of stress legacy in clonal transgenerational effects: A case study on Trifolium repens
Source: Ecol Evol. 2022 May 24;12(5):e8959. doi: 10.1002/ece3.8959 (PMC9130644; doi:10.1002/ece3.8959)
Supplement: Supplementary file 2 — Fig S2 [file ECE3-12-e8959-s003.pdf]

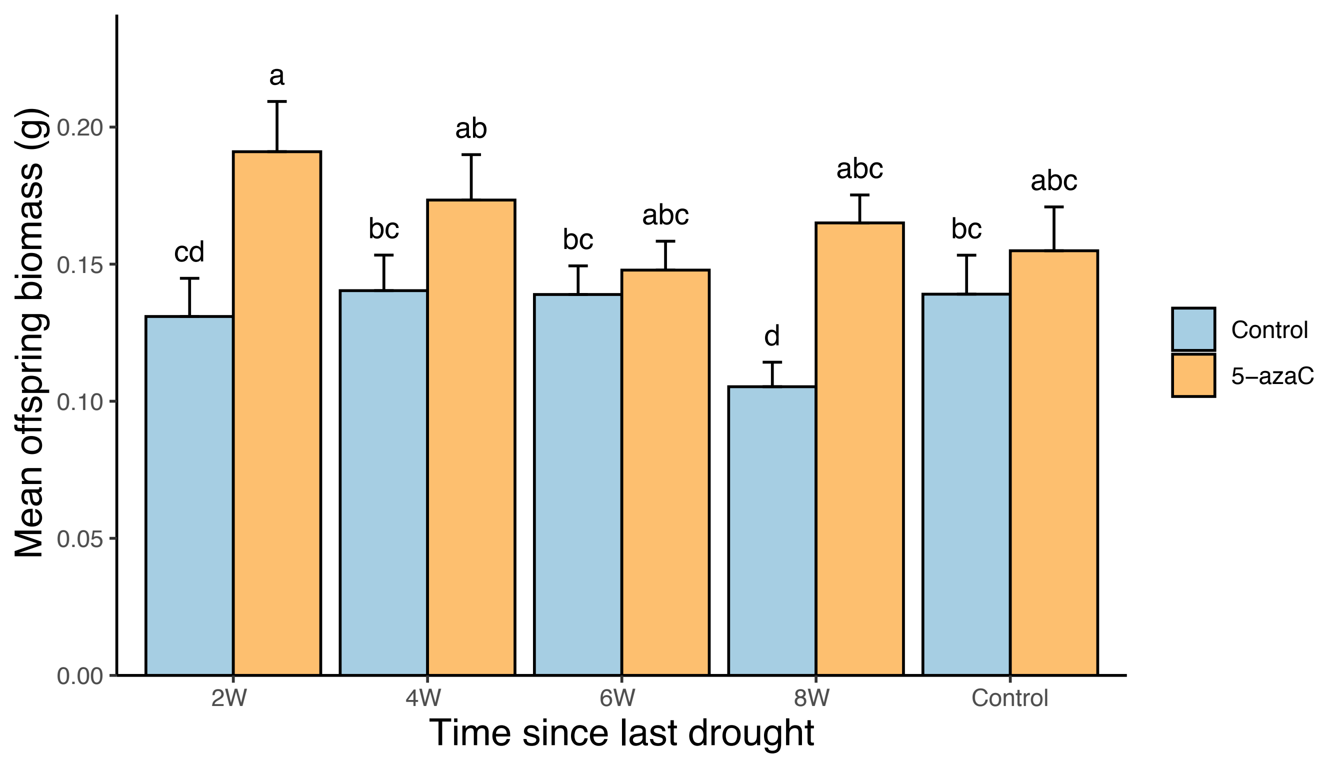
Mean offspring biomass (g)
0.00
0.05
0.10
0.15
0.20
cd
a
bc
ab
bc
abc
d
abc
bc
abc
2W
4W
6W
8W
Control
Time since last drought
Control
5−azaC
