# Supplementary material for: Time dynamics of stress legacy in clonal transgenerational effects: A case study on Trifolium repens
Source: Ecol Evol. 2022 May 24;12(5):e8959. doi: 10.1002/ece3.8959 (PMC9130644; doi:10.1002/ece3.8959)
Supplement: Supplementary file 9 — Table S1–S5 [file ECE3-12-e8959-s006.docx]

Table S1 AIC value of alternative models explaining parental biomass, mean offspring biomass developed in the second phase and final side branch number. The optimal model (with lowest AIC) is in bold.

| Models | parental biomass | mean offspring biomass | side branch no. |
| --- | --- | --- | --- |
| (genotype + time since last drought + methylation) | **-6.12** | 70.85 | **601.63** |
| (genotype + time since last drought + methylation)^2 | 0.92 | 68.94 | 612.56 |
| (genotype + time since last drought + methylation)^3 | 2.42 | **64.05** | 625.63 |
| (genotype+ time since last drought +methylation + initial size) | - | 71.81 | 603.52 |
| (genotype+ time since last drought +methylation + initial size)^2 | - | 72.89 | 622.61 |
| (genotype+ time since last drought +methylation + initial size)^3 | - | 85.1 | 655.5885 |

Table S2. Estimate of parameters from the model testing the effects of genotype, time since the last drought (2W, 4W, 6W, 8W and Control) and 5-azaC treatment (control *versus* 5-azaC) on parental biomass, mean offspring biomass developed in the second phase and final side branches number.

|  | Parental biomass | mean offspring biomass | side branch no. |
| --- | --- | --- | --- |
| (Intercept) | 3.989 | -2.227 | 2.095 |
| genotypeB | -0.340 | 0.420 | 0.012 |
| genotypeC | -0.011 | 0.193 | -0.208 |
| 2W | -1.422 | 0.487 | 0.310 |
| 4W | -1.265 | 0.117 | 0.543 |
| 6W | -1.251 | 0.231 | 0.354 |
| 8W | -1.035 | 0.024 | 0.279 |
| 5-Aza | 0.063 | 0.206 | -0.226 |
| genotypeB:2W | - | -0.844 | - |
| genotypeC:2W | - | -0.821 | - |
| genotypeB:4W | - | -0.319 | - |
| genotypeC:4W | - | -0.006 | - |
| genotypeB:6W | - | -0.484 | - |
| genotypeC:6W | - | -0.142 | - |
| genotypeB:8W | - | -0.357 | - |
| genotypeC:8W | - | -0.535 | - |
| genotypeB:5-Aza | - | -0.383 | - |
| genotypeC:5-Aza | - | 0.072 | - |
| 2W:5-Aza | - | -0.346 | - |
| 4W:5-Aza | - | 0.287 | - |
| 6W:5-Aza | - | -0.181 | - |
| 8W:5-Aza | - | 0.073 | - |
| genotypeB:2W:5-Aza | - | 1.197 | - |
| genotypeC:2W:5-Aza | - | 0.699 | - |
| genotypeB:4W:5-Aza | - | -0.147 | - |
| genotypeC:4W:5-Aza | - | -0.388 | - |
| genotypeB:6W:5-Aza | - | 0.278 | - |
| genotypeC:6W:5-Aza | - | 0.144 | - |
| genotypeB:8W:5-Aza | - | 0.399 | - |
| genotypeC:8W:5-Aza | - | 0.500 | - |

Table S3 AIC value of alternative models explaining side branch number, node number and stolon length developed in the second phase. The optimal model (with lowest AIC) is in bold. – indicates a model that did not converge and thus could not be included in the comparison.

| Models | side branch no. | node number | stolon length |
| --- | --- | --- | --- |
| (genotype + time since last drought + methylation + time of measurement) | 3934.25 | 5429.42 | 3766.5 |
| (genotype + time since last drought + methylation + time of measurement)^2 | 3885.61 | **5420.24** | 3154.58 |
| (genotype + time since last drought + methylation + time of measurement)^3 | 3867.62 | - | 3077.19 |
| (genotype+ time since last drought +methylation+ time of measurement + initial size) | 3934.66 | 5430.84 | 3766.63 |
| (genotype+ time since last drought +methylation+ time of measurement + initial size)^2 | 3875.53 | - | 3110.04 |
| (genotype+ time since last drought +methylation+ time of measurement + initial size)^3 | **3849.13** | - | **2933.7** |

Table S4 Effects of genotype, time since last drought (2W, 4W, 6W,8W and Control) and 5-azaC treatment (control *versus* 5-azaC), time of measurement (T1 to T10) and initial size on side branch number and stolon length developed in the second phase based on the optimal model. Values for P < 0.05 are in bold. Marginally significant (P < 0.1) in italics.

|  | DF | Side branch no. | | Stolon length | |
| --- | --- | --- | --- | --- | --- |
|  |  | F value | Pr(Chi) | F value | Pr(Chi) |
| genotype | 2 | 4.51 | **<0.001** | 0.91 | 0.408 |
| day (time since last drought) | 4 | 7.94 | **<0.001** | 0.31 | 0.873 |
| methylation | 1 | 17.58 | **<0.001** | 1.09 | 0.300 |
| time (time of measurement) | 1 | 2439.24 | **<0.001** | 590.27 | **<0.001** |
| initial size | 1 | 1.57 | 0.486 | 6.35 | **0.014** |
| genotype×day | 8 | 1.43 | **0.019** | 0.35 | 0.941 |
| genotype×methylation | 2 | 0.47 | 0.218 | 0.02 | 0.983 |
| genotype×time | 2 | 6.87 | **<0.001** | 71.63 | **<0.001** |
| genotype×initial size | 2 | 6.83 | 0.198 | 0.81 | 0.448 |
| day×methylation | 4 | 3.69 | **0.004** | 0.54 | 0.704 |
| day×time | 4 | 12.58 | **<0.001** | 17.88 | **<0.001** |
| day×initial size | 4 | 3.63 | 0.049 | 0.27 | 0.896 |
| methylation×time | 1 | 0.01 | 0.879 | 4.34 | **0.038** |
| methylation×initial size | 1 | 1.00 | 0.326 | 0.85 | 0.360 |
| time×initial size | 1 | 11.23 | **0.002** | 90.49 | **<0.001** |
| genotype×day×methylation | 8 | 0.89 | 0.743 | 0.75 | 0.649 |
| genotype×day×time | 8 | 1.61 | 0.113 | 8.72 | **<0.001** |
| genotype×day×initial size | 8 | 1.09 | 0.461 | 0.51 | 0.846 |
| genotype×methylation×time | 2 | 0.40 | 0.996 | 5.72 | **0.003** |
| genotype×methylation×initial size | 2 | 0.07 | 0.947 | 0.01 | 0.989 |
| genotype×time×initial size | 2 | 3.88 | 0.140 | 31.62 | **<0.001** |
| day×methylation×time | 4 | 3.55 | **0.050** | 21.05 | **<0.001** |
| day×methylation×initial size | 4 | 0.34 | 0.852 | 0.56 | 0.691 |
| day×time×initial size | 4 | 2.75 | *0.068* | 9.53 | **<0.001** |
| methylation×time×initial size | 1 | 0.59 | 0.439 | 2.76 | *0.097* |

Table S5 Effects of genotype, time since last drought (2W, 4W, 6W,8W and Control) and 5-azaC treatment (control *versus* 5-azaC), and time of measurement (T1 to T10) based on the optimal model on node number developed in the second phase. Values for P < 0.05 are in bold. Marginally significant (P < 0.1) in italics.

|  | DF | node number | |
| --- | --- | --- | --- |
|  |  | F value | Pr(Chi) |
| genotype | 2 | 23.35 | **<0.001** |
| day (time since last drought) | 4 | 7.27 | **0.001** |
| methylation | 1 | 0.24 | 0.537 |
| time (time of measurement) | 1 | 1015.69 | **<0.001** |
| genotype×day | 8 | 1.37 | 0.223 |
| genotype×methylation | 2 | 0.14 | 0.838 |
| genotype×time | 2 | 7.56 | **<0.001** |
| day×methylation | 4 | 3.26 | **0.015** |
| day×time | 4 | 4.03 | **0.003** |
| methylation×time | 1 | 0.60 | 0.439 |
